# Transcriptional programming of immunoregulatory responses in human Langerhans cells

**DOI:** 10.3389/fimmu.2022.892254

**Published:** 2022-09-20

**Authors:** James Davies, Sofia Sirvent, Andres F. Vallejo, Kalum Clayton, Gemma Douilhet, Patrick S. Keeler, Jonathan West, Michael Ardern-Jones, Ben D. MacArthur, Harinder Singh, Marta E. Polak

**Affiliations:** ^1^ Clinical and Experimental Sciences, Sir Henry Wellcome Laboratories, Faculty of Medicine, University of Southampton, Southampton, United Kingdom; ^2^ Human Development and Health, Faculty of Medicine, University of Southampton, Southampton, United Kingdom; ^3^ Cancer Sciences, Faculty of Medicine, University of Southampton, Southampton, United Kingdom; ^4^ Institute for Life Sciences, University of Southampton, Southampton, United Kingdom; ^5^ Center for Systems Immunology, Departments of Immunology and Computational and Systems Biology, The University of Pittsburgh, Pittsburgh, PA, United States

**Keywords:** dendritic cell (DC), Langerhans cell (LC), gene regulatory network (GRN), transcriptional regulation, immune regulation

## Abstract

Human epidermal Langerhans cells (LCs) maintain immune homeostasis in the skin. To examine transcriptional programming of human primary LCs during homeostasis, we performed scRNA-seq analysis of LCs before and after migration from the epidermis, coupled with functional assessment of their regulatory T cell priming capabilities. The analysis revealed that steady-state LCs exist in a continuum of maturation states and upregulate antigen presentation genes along with an immunoregulatory module including the genes *IDO1*, *LGALS1*, *LAMTOR1, IL4I*, upon their migration. The migration-induced transition in genomic state is accompanied by the ability of LCs to more efficiently prime regulatory T cell responses in co-culture assays. Computational analyses of the scRNAseq datasets using SCENIC and Partial Information Decomposition in Context identified a set of migration-induced transcription factors including IRF4, KLF6 and RelB as key nodes within a immunoregulatory gene regulatory network. These findings support a model in which efficient priming of immunoregulatory responses by LCs is dependent on coordinated upregulation of a migration-coupled maturation program with a immunoregulation-promoting genomic module.

## Introduction

Langerhans cells (LCs) reside in the epidermis as a dense network of immune system sentinels, capable of initiating potent immune responses to cutaneous pathogens and neoplastic cells (1–3). As a first line of the cutaneous immune defense system, LCs are uniquely specialized at sensing the environment by extending dendrites through inter-cellular tight junctions to gain access to the outermost part of the skin, the stratum corneum, so that rapid responses can be initiated if a dangerous pathogen is encountered (4). We and others have shown that LCs are capable of priming CD4 T cell responses and can also activate CD8 T cells *via* antigen cross-presentation, the latter more effectively than CD14+ and CD11c+ dermal dendritic cells (DCs) (5–8). In contrast, during steady-state conditions (non-pathogenic contexts), LCs induce the activation and proliferation of skin-resident regulatory T cells (9, 10) that prevent unwanted immune-mediated pathology. Recent analysis of single cell transcriptional programmes uncovered *in situ* heterogeneity of human steady-state LCs across different body sites, and suggested the role of PU.1, ID2 and NFkB transcription factors in controlling LC differentiation and activation (11). In the healthy skin LC migration from the epidermis, occurring during non-inflammatory conditions, involves transport of self-antigens, such as those derived from melanin, to skin draining lymph nodes and the priming of tolerogenic regulatory T cells (Tregs) (12–14). In accord with these findings, in a mouse model of autoimmune encephalomyelitis, migratory skin LCs and not lymphoid-resident resident DCs have been shown to induce Foxp3+ Tregs and improve disease outcome (15). While migratory LC appear to play important roles in immunoregulation and cutaneous homeostasis, the nature of the transcriptional states underlying migratory LC immunoregulatory responses remain to be elucidated.

A long standing and widely-held view posits that priming of immunoregulatory responses is a consequence of the immature status of antigen presenting cells (APCs), including LCs and DCs in peripheral tissues (16–20). The immature status of APCs is associated with lower levels of antigen-processing and presentation components as well as co-stimulatory molecules required for efficient T cell priming and activation. According to this view, maturation of APCs that accompanies their migration to draining lymph nodes is necessary for efficient priming of immunogenic but not tolerogenic T cell responses. However, DC maturation has also been shown to occur under steady-state conditions, in particular by activation of the β-catenin pathway and the disruption of E-cadherin mediated adhesion (21). E-cadherin-stimulated DCs undergo maturation with the upregulation of MHC class II, co-stimulatory molecules and chemokine receptors and prime regulatory T cells responses that that suppress immune responses *in vivo* thereby promoting tolerance. Consistent with these findings, Wnt-β-catenin signalling in intestinal dendritic cells has been shown to be required for expression of anti-inflammatory mediators including retinoic acid-metabolizing enzymes and interleukin-10 and the stimulation of Treg induction (22). Accordingly, loss of β-catenin in DCs results in enhanced inflammatory bowel disease in a mouse model (22).

Although the concept linking the pathogen induced migration and maturation of APCs with their preferential priming of immunogenic responses is consistent with the largely immature status of steady-state LCs (7, 8, 23) and the observations that such LCs can activate skin resident regulatory T (Treg) cells (9, 24), it is challenged by the following. As with DCs, recent findings suggest that the induction and maintenance of cutaneous homeostasis is dependent on LC migration to the lymph nodes (12–14). Notably, such non-inflammatory migratory LCs express significantly higher levels of MHC II and co-stimulatory molecules (7, 8, 25), thereby implicating the importance of LC maturation for the efficient immunoregulation.

Using an *in vitro* system involving murine bone-marrow derived DCs, we have previously shown that in the absence of pathogen derived signals, the transcription factor interferon regulatory factor 4 (IRF4) promotes the expression of genes required for antigen presentation along with those for T cell tolerance, thus enabling the efficient priming of Treg responses (26). Furthermore, we have recently demonstrated that the migration of human LCs is associated with the upregulation of IRF4 and the enhanced expression of MHC-II complexes and co-stimulatory molecules required for T cell activation (8, 27). The IRF4 dependent gene regulatory network has been proposed to counterbalance the induction of LC activation by pro-inflammatory cytokines coordinated by IRF1 (8, 28). Here we sought to compare the transcriptional programming of steady-state human LCs with their migratory counterparts so as to test our regulatory framework and to gain further insight into transcriptional regulatory circuits that could couple LC maturation with the functional priming of regulatory T cell responses.

Utilizing scRNA-Seq we reveal key differences in transcriptional programming of non-inflammatory LCs. Upon migration, LCs induced the expression of an immunoregulatory gene module including the *IDO1*, *LGALS1*, *LAMTOR1*, *IL10RA* and *IL4L1* genes and this was accompanied with their more efficient priming of regulatory T cell responses in co-culture assays. Regulon-focused analyses of transcriptional programmes, including Partial Information Decomposition in Context, identified IRF4 along with KLF6 and RELB as key regulators of the immunoregulatory program. Select predictions were validated by analysis of CRISPR-Cas9 IRF4-edited LCs. Our findings support the concept that efficient priming of immunoregulatory responses by LCs under non-inflammatory conditions requires upregulation of a migration-induced maturation program coupled with an immunoregulation-inducing gene module.

## Materials and methods

### Human LC, TRM and PBMC isolation

Human blood and skin mastectomy and abdominoplasty samples were collected with written consent from donors with approval by the South East Coast - Brighton & Sussex Research Ethics Committee in adherence to Helsinki Guidelines [ethical approvals: REC approval: 16/LO/0999). Fat and lower dermis was cut away and discarded before dispase (2 U/ml, Gibco, UK, 20h, +4°C) digestion. For steady-state LC extraction, epidermal sheets were digested in Liberase™ (13 U/ml, Roche, UK, 2h, +37°C), and enriched using density gradient centrifugation (Optiprep 1:4.2, Axis Shield, Norway). Migrated LCs and TRMs were extracted from epidermal explant sheets cultured in media (RPMI, Gibco, UK, 5%FBS, Invitrogen, UK, 100 IU/ml penicillin and 100 mg/ml streptomycin, Sigma, UK) following migration for 48 hours. MigLCs were enriched using density gradient centrifugation (Optiprep 1:4.2, STEMCELL, UK). TRMs were purified using density gradient separation (volumes 1:3, Optiprep, STEMCELL, UK). Steady state and migLC were processed through fluorescence-activated cell sorting (FACS) as described below and processed for Drop-seq or cryopreserved in 90% FBS (Gibco, UK), 10% DMSO (Sigma, UK). PBMCs were extracted from human blood using lymphoprep (Stemcell, UK) density gradient separation. Naïve T cells were purified using the Naïve CD4+ T cell isolation kit (Miltenyi Biotec, UK).

### Flow cytometry/FACS

Antibodies used for cell staining were pre-titrated and used at optimal concentrations. A FACS Aria flow cytometer (Becton Dickinson, USA) and FlowJo software was used for analysis. For FACS purification LCs were stained for CD207 (anti-CD207 PeVio700), CD1a (anti-CD1a VioBlue) and HLA-DR (anti-HLA-DR Viogreen, Miltenyi Biotech, UK). For T cell staining, antibodies anti-CD3 PerCP, anti-CD4 Viogreen, anti-CD127 Pe (Miltenyi Biotech, UK) and anti-CD25 PeCy7 (Invitrogen, UK) were used for surface staining. Anti-FOXP3 FITC (eBiosciences, UK), anti-IL-10 PE (Miltenyi, UK) and anti-IDO1 AlexaFluor647 (Biolegend, UK) antibodies were used for intranuclear and intracellular staining. IDO1 intracellular staining of LCs was performed using Intracellular Fixation & Permeabilization Buffer Set (eBioscience, UK), following manufacturer protocol.

### Co-culture, suppression and inhibition assays

For co-culture assays, purified LC and naïve CD4+ T cells or TRMs were co-cultured in human serum supplemented media (RPMI, Gibco, UK, 10% human serum, Sigma, UK, 100 IU/ml penicillin and 100 mg/ml streptomycin, Sigma, UK) at a 1:50 ratio for 5-days at 37°C. For intranuclear FOXP3 staining T cells were permeabilised using the FOXP3/Transcription Factor Staining Buffer Set (eBiosciences, UK) following the manufacturers protocol, after cell surface marker staining. For IL-10 intracellular staining, T cells were stimulated with cell stimulation cocktail (eBioscience, UK) for 6 hours and Golgi plug (eBioscience, UK) for 5 hours, prior to intracellular staining using Permeabilizing Solution 2 (BD Biosciencies, UK). To measure proliferation, PBMCs were labelled with CFSE using the CellTrace™ CFSE Cell Proliferation Kit (Invitrogen, UK), with ice cold PBS, 0.5% BSA replacing PBS and ice cold media replacing pre-warmed media as described in the protocol. Proliferation inhibition assays were set up through supplementing CFSE labelled PBMCs with autologous FACS-purified CD3+CD4+CD127-CD25+ T cells induced after 5-day naïve CD4+ T cells and FACS-purified LC co-cultures at 1:1 and 1:3 ratio. Proliferation was assessed by flow cytometry on day 3 of co-cultures.

### Drop-seq

After FACS purification, single LCs were co-encapsulated with primer coated barcoded Bead SeqB (Chemgenes, USA) within 1 nL droplets [Drop-seq (29)]. Drop-seq microfluidic devices according to the design of Macosko et al. were fabricated by soft lithography, oxygen plasma bonded to glass and functionalised with fluorinated silane (1% (v/v) trichloro(1H,1H,2H,2H-*perfluorooctyl)silane* in HFE-7500 carrier oil). Open instrumentation syringe pumps and microscopes (see dropletkitchen.github.io) were used to generate and observe droplets, using conditions and concentrations according to the Drop-seq protocol. Purified LCs were converted into ‘STAMPs’ for PCR library amplification (High Sensitivity DNA Assay, Agilent Bioanalyser) and tagmentation (Nextera XT, Illumina, UK). Sequencing of libraries was executed using NextSeq on a paired end run (1.5x10E5 reads for maximal coverage) at the Wessex Investigational Sciences Hub laboratory, University of Southampton.

### scRNASeq data analysis

The Drop-seq protocol from the McCarrol lab (29) was followed for converting sequencer output into gene expression data. Briefly, the bcl2fastq tool from Illumina was used to demultiplex files, remove UMIs from reads and deduce captured transcript reads. Reads were then aligned to human hg19 reference genome using STAR. Analyses was performed using the python-based Scanpy pipeline (version 1.5.0) (30). High quality barcodes, discriminated from background RNA barcodes, were selected based on the overall UMI distribution using EmptyDrops (31). Low quality cells, with a high fraction of counts from mitochondrial genes (20% or more) indicating stressed or dying cells were removed. In addition, genes with expression detected in <10 cells were excluded. Datasets were normalised using Scran (32). Highly variable genes (top 2000) were selected using distribution criteria: min_mean=0, max_mean=4, min_disp=0.1. A single-cell neighbourhood graph was computed on the first principal components that sufficiently explain the variation in the data using 10 nearest neighbours. Uniform Manifold Approximation and Projection (UMAP) was performed for dimensionality reduction. Leiden algorithm (33) was used to identify clusters within cell populations (Leiden r = 0.5, n_pcs=30). Differentially expressed genes (DEGs) between cell clusters were identified using T-test (FDR corrected p-value<0.01, logFC>1). Gene ontology analysis was performed using Toppgene (34) (FDR corrected p-value<0.05), describing biological pathways associated with gene lists. Gene signature enrichment was performed using Gene Set Variation Analysis (GSVA) (35). Regulatory network inference analysis was performed using single-cell regulatory network inference and clustering (pySCENIC) (36).

### Reanalysis of skin antigen presenting data from public domain

Public datasets from GEO used for defining tolLC signature included a microarray dataset containing dexamethasone and vitamin D3 stimulated MoDC (TolMoDC) with unstimulated MoDC (GSE52894) and a microarray dataset containing trypsinised steady-state LC with unstimulated MoDC (GSE23618) Normalized count matrices were downloaded from GEO before Limma DEG analysis. DEGs upregulated in LCs and TolMoDCs compared to unstimulated MoDCs from each respective dataset were anlaysed, with unstimulated MoDC used as reference for comparison. A microarray dataset, of migratory LC, CD14+ DDC, CD141+ DDC and CD14-CD141- DDC (GSE66355 (5), exported from GEO (Gene Expression Omnibus), was background corrected and quantile normalised using Limma (37) within an R environment. 25/30 of the Tolerogenic DC signature 1 genes expressed in LC single cell RNA-seq data, were expressed in the microarray dataset and were plotted as heatmaps using the gplots package.

**Figure 1:**
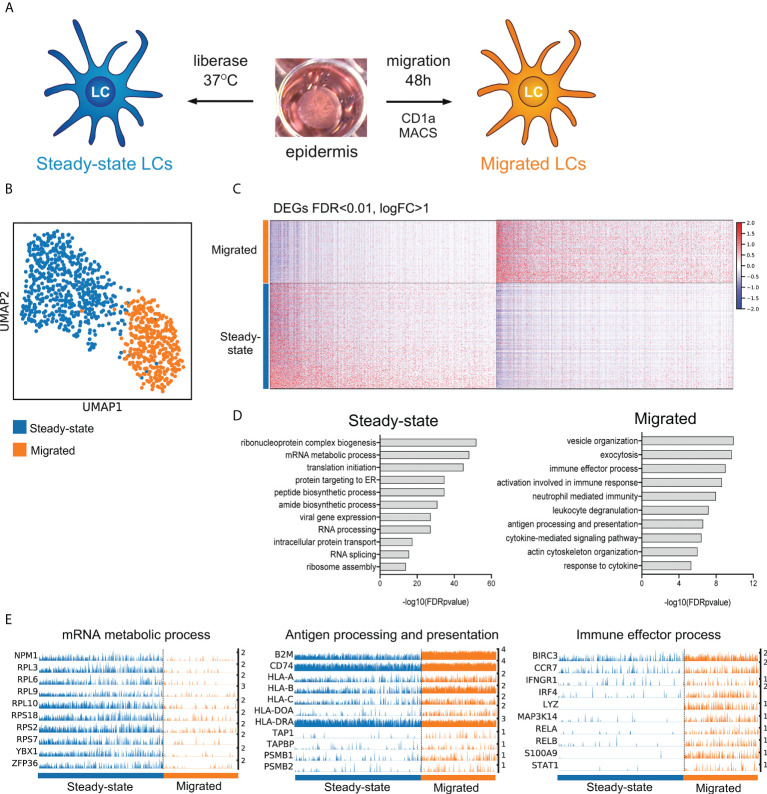
LC migration from the epidermis induces immunocompetence associated transcriptional modules. **(A)** A schematic illustrating isolation of primary human LCs. Split healthy skin was treated with dispase for 20 h to dissociate epidermis. Steady-state LCs were isolated from the epidermis by digestion with liberase TM or migrated from the epidermal sheets for 48 h in cell culture medium **(B)** UMAP dimensionality reduction analysis of Scran normalised single cell data from steady-state (585) and migrated (387) LCs originating from the same donor. **(C)** Heatmap displaying the 1002 upregulated DEGs in steady-state LC and 1012 DEGs upregulated in migrated LC (FDR corrected p=<0.01, logFC>1). **(D)** Gene ontology analysis (Toppgene) results are displayed alongside for steady-state and migrated LC upregulated DEGs (-log10 FDR corrected p-values) **(E)** Trackplots displaying genes included in ontologies upregulated in steady-state (mRNA metabolic process) and migrated LC (antigen processing and presentation, immune effector process).

For tracking signatures across single cell transcriptomes from skin antigen presenting cells myeloid cell transcriptome data were downloaded from Reynolds et al. (38) and were processed from normalized data downloaded from the Zenodo repository (ID: 4536165). The annotation from the original publication was used for sub-setting antigen presenting cells. UMAP (39) (v 0.5.2) and leiden (33) (v 0.8.8) were applied to detect the LC populations (50 PCs and 10 neighbourghs, r=0.5). Signature overlaps were carried out following sample QC and normalisation in ScanPy (v 1.8.2), using Jupyter Notebook in Google Colab environment. LC1, LC2, actLC and migLC markers from Liu et al. (11). (**Figure 1B** heatmap gene lists) were plotted as individual expression values, or as average expression of whole signatures (z-scores), in LC single cell RNA-seq data obtained in-house.

### Directional PIDC

Notebooks from Chan et al. (40) were adapted for the analysis and run using Julia V 1.0.5 in Jupyter Notebook. SCRAN-normalised data for migrated LCs including genes from tol1 signatures and selected transcription factors was used for network inference using PDIC algorithm. Edge weights were exported and sorted to include only transcription factors as targets. Hierarchical network was visualised using yED.

## Results

### LC migration from the epidermis induces immunocompetence associated transcriptional modules

To analyze the transcriptional states of steady-state and migrated LCs we performed scRNA-Seq on LC dissociated from healthy skin using the dispase/liberase protocol and after their migration from the epidermis **Figure 1A** (8). The single-cell transcriptome analyses revealed a dramatic switch in transcriptional programmes following LC migration out of the epidermis, when LCs from the same donor (**Figures 1B–E**), or multiple donors (**Supplementary Figures S1 A-C**) were contrasted. UMAP dimensionality reduction analysis of the dataset revealed distinct clustering of steady-state and migrated LCs (**Figure 1B**, **Supplementary Figures S1A**). Analysis of differentially expressed genes (Steady-state=1002 upregulated genes, migrated=1012 upregulated genes, **Supplementary Table 1**, **Figure 1C**) strongly suggested that LC migration induced their maturation and immunocompetence, reflected by the increased expression of genes involved in antigen processing and presentation (p=2.5E-7) as well as induction of immune effector processes (p=9.8E-10, **Figures 1D, E** and **Supplementary Figures 1C, D**). Interestingly, UMAP dimensionality reduction analysis followed by Leiden clustering identified two subpopulations of steady-state LCs, S1 and S2 (**Supplementary Figure 1D**). Contrasting gene expression between the two subpopulations identified 372 upregulated genes in S2, indicative of a poised state for activation of adaptive immune responses (**Supplementary Figures 1E–H**, **Supplementary Table 2**). When compared with recently described scRNA-seq datasets of human LC subsets (11), S1 cluster cells more strongly resembled LC1 expressing *EPCAM*, *HPGDS*, *PRKCB* and *CSF1R*, while LC2 specific genes, except for *IL1B*, were expressed comparably between steady-state and migrated LCs. Both S1 and S2 expressed genes involved in actLC transcriptional state, including *PIM3*, *MMP9*, *LMNA* and *STK17B* (**Supplementary Figures 1 I–J**).

### Migration of LCs from the epidermis enhances their immunoregulatory transcriptional programming

**Figure 2:**
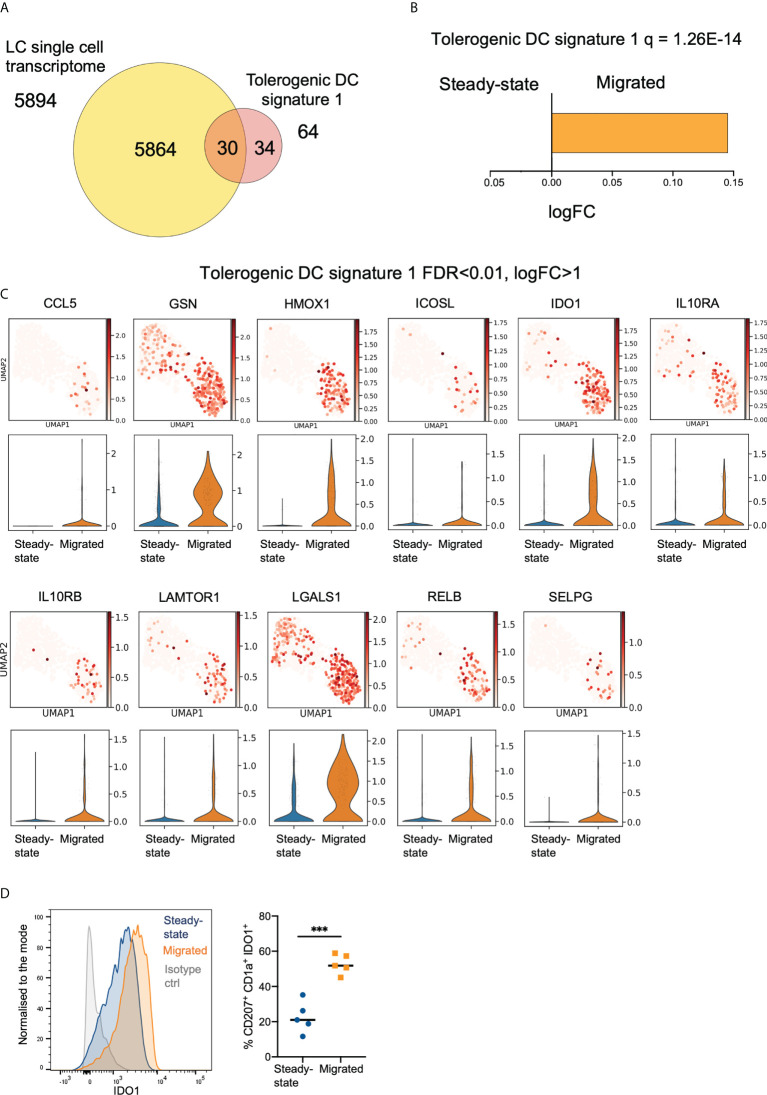
Migration of LCs from the epidermis enhances their immunoregulatory transcriptional programming. **(A)** Venn diagram displaying the number of genes from tolerogenic gene signature 1 (tol 1), curated from literature exploring genes associated with DC or macrophage tolerogenic function, within the whole LC single cell dataset. **(B)** Gene Set Variation Analysis (GSVA) displaying enrichment of tol 1 in the LC populations. FDR corrected p-values and logFC are displayed. **(C)** Violin plots and UMAP marker plots displaying the expression of genes within tol 1 amongst steady-state and migrated LCs (FDR corrected p-values <0.01, logFC>1). **(D)** Flow cytometry analysis of IDO1 protein expression in steady-state and migrated LC extracted by 48 hour culture of epidermal sheets. n=5 steady-state and migrated independent LCs versus isotype control (grey), n=4 migrated LCs. ***p<0.001.

To investigate transcriptional programmes underlying the ability of LCs to induce immunoregulation, we assembled two tolerogenic DC gene signature panels, Tol1 (**Figure 2A**, **Supplementary Figure 2A**, **Supplementary Table 3**) and Tol2 (**Supplementary Figures 2B–D**, **Supplementary Table 4**) based on previous studies (GSE23618, GSE52850, GSE52894, GSE117946). The Tol2 signature contained 217 common genes co-upregulated in two or more of the tolerogenic DC conditions: monocyte-derived DCs (MoDC) exposed to VitD3, Dexamethasone and IL10, and DCs isolated from placenta, a well-known immune privileged site, when contrasted with unstimulated MoDCs. Notably, both Tol1 and Tol2 signatures were significantly enriched in our LC datasets, (Tol1 = 1.26E-14, Tol2 = 1.45E-9), with LCs expressing 30/64 genes of the Tol1 signature (**Figure 2A, B**), and 112/217 genes of the Tol 2 signature (**Supplementary Figures 2E, F**). Importantly, both signatures were more highly manifested in migrated LCs, in comparison with steady state LCs (**Figures 2B, C**, **Supplementary Figures 2E–G**). Similarly, migLCs showed the highest levels of expression of 30 of the 64 Tol1 genes when compared with CD14+, CD141+ and CD141/CD14- migratory dermal dendritic cell populations reported by Artyomov et al. (5) (**Supplementary Figure 2H**). We confirmed the overexpression of Tol1 genes in LC from healthy skin in comparison to different skin antigen presenting cell subsets in the single cell skin atlas (38) (**Supplementary Figures 2I, J**), and delineated enrichment of genes associated with mregDC transcriptional programme in LCs from healthy skin (41) (**Supplementary Figures 2I, K**). Consistent with the scRNA-Seq data, expression of a hallmark immunoregulatory protein, Indoleamine 2,3-dioxygenase 1 (IDO1) was considerably higher in migrated LCs compared to steady-state LC (p<0.001, **Figure 2D**).

### Migrated LCs more efficiently prime functional Treg responses

**Figure 3:**
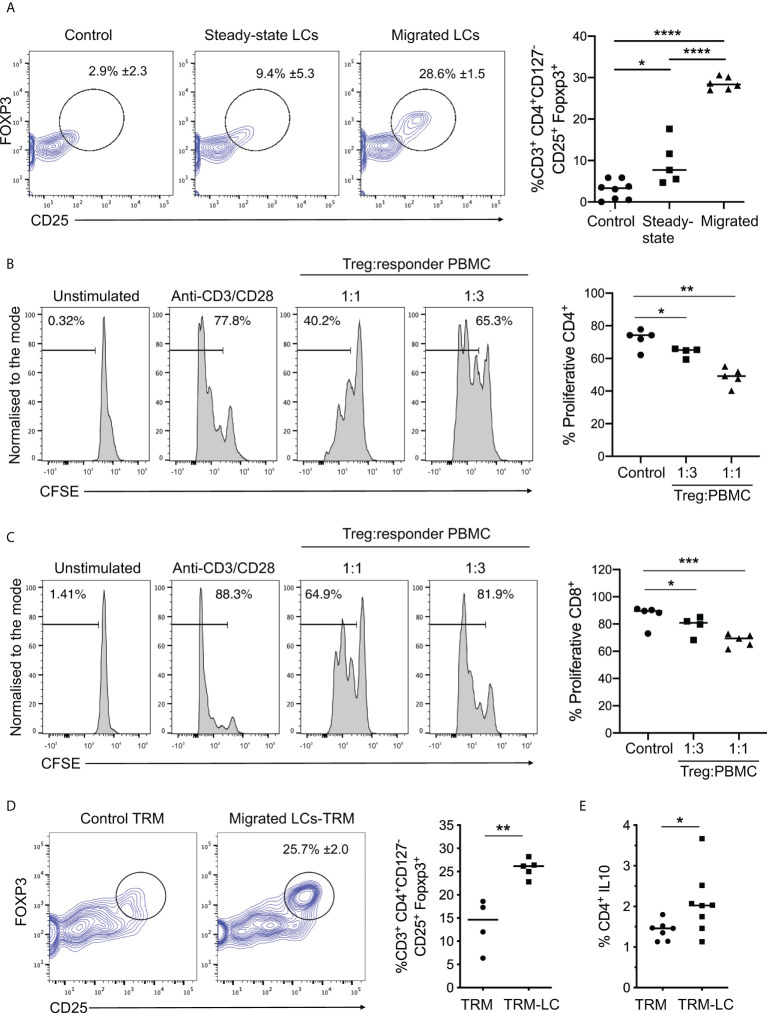
Migrated LCs more efficiently prime functional Treg responses. **(A)** Flow cytometry analysis of Tregs induced after co-culture of steady-state and migrated LC with CD4+ naive T cells as in Fig. **2C**. n=8 control, n=5 steady-state LCs and n=6 migrated LCs from independent donors. *p<0.05, **p<0.01, ***p<0.001. **(B)** Proliferation analysis of CD4+ T cells using CFSE labelled PBMCs after 3-day co-culture with autologous purified CD3+CD4+CD127-CD25+ Tregs. The percentages of proliferating CD4+ cells stimulated with plate bound anti-CD3 and soluble anti-CD28 are displayed at ratios of 1:1 and 1:3 Treg : PBMC (n=5 from 3 independent LC donors). *p<0.05, **p<0.01. **(C)** Proliferation analysis of CD8+ T cells using CFSE labelled PBMCs after 3-day co-culture with autologous purified CD3+CD4+CD127-CD25+ Tregs. The percentages of of proliferating CD8+ cells stimulated with plate bound anti-CD3 and soluble anti-CD28 are displayed at ratios of 1:1 and 1:3 Treg : PBMC (n=5 from 3 independent LC donors). *p<0.05, ***p<0.001. **(D)** Flow cytometry assessment of the percentage of Tregs induced after 5-day co-culture of migrated LC with autologous TRMs extracted from human epidermis. 5-day cultures of TRMs alone were used as control. Tregs were identified as CD3+CD4+CD127-CD25+FOXP3+ cells. n=5 independent LC donors. **p<0.01. **(E)** Percentage of IL-10 producing CD4+ cells after co-culture of TRMs in the presence or absence of migrated LC. n=8. *p<0.05, ****p<0.0001.

To explore the linkage between increased LC immunocompetence and induction of immunoregulation suggested by the scRNA-seq analysis, we examined the effect of LC migration on their ability to prime functional Treg responses. To do so, we measured the induction of CD25+FOXP3+ Tregs by LCs following their 5-day co-culture with naïve CD4+ T cells (**Figure 3A**). Migrated LC induced significantly higher frequencies of CD25+FOXP3+ Tregs compared to their steady-state counterparts (**Figure 3A**, **Supplementary Figures 3A-C**). Importantly, Tregs induced by migrated LCs inhibited CD4 and CD8 T cell proliferation (**Figures 3B, C**, respectively). Thus, migrated LCs have enhanced potential for inducing functional Tregs, and such cells can suppress activated CD4 and CD8 T cell responses. Furthermore, co-culture of migrated LCs with tissue resident memory T cells (TRMs) significantly increased the number of CD25+FOXP3+ Tregs compared to steady-state controls (**Figure 3D**, n=5 steady-state LC independent skin donors, n=4 migrated LC independent skin donors, p=0.0025). Notably, co-culture of migrated LCs with resident memory T cells also promoted expansion of IL-10 producing CD4+ T cells (**Figure 3E**, n=8 independent skin donors, p=0.0451) and enhanced TRM viability (**Supplementary Figure 3D**). Thus migrated LCs are more efficient at priming both naïve and memory Treg responses. Corroborating the importance of LC maturation for induction of regulatory responses, CD86high S2 LCs expanded CD25+FOXP3+ Tregs more efficiently than CD86 low S1 LCs (**Supplementary Figures 3E–G**).

### Transcriptional network underlying LC immunoregulatory programming

**Figure 4:**
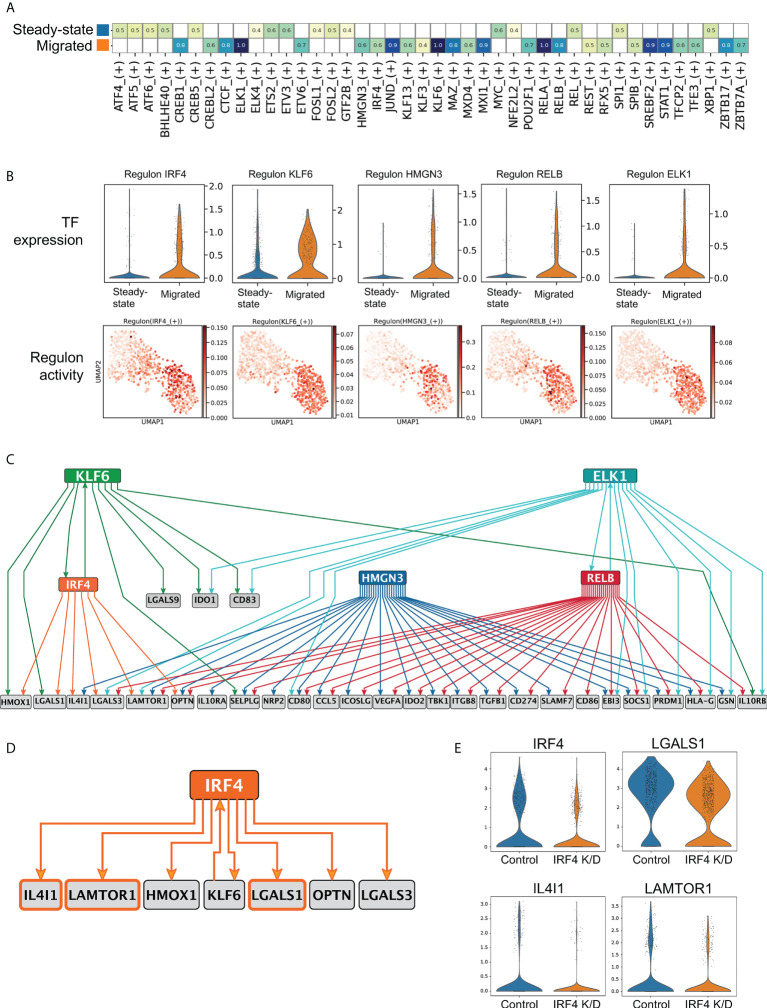
Transcriptional network underlying LC immunoregulatory programming. **(A)** SCENIC regulatory network and inference clustering analysis revealed TF regulons which were enriched in steady-state and migrated LCs from the same donor. Z-score heatmap of enriched regulons are displayed (z-score>0.4). **(B)** Violin plots displaying the transcriptomic expression of TFs identified to be enriched in migrated LCs from SCENIC analysis. UMAP marker plots showing TF regulon enrichment Z-scores in each cell, across the two LC populations are displayed. **(C)** PIDC network graph comprising 70 edges with weight >1, hierarchically organized, displaying predicted regulatory modules for the top 5 enriched TFs with genes within the tol 1 signature. **(D)** PIDC Network displaying IRF4 with 6 target genes and 1 transcription factor as predicted by PIDC. **(E)** Violin plots displaying the 3 predicted *IRF4* regulated genes (*IL4I1, LGALS1, LAMTOR1*) that were identified to be downregulated in CRISPR-Cas9 IRF4 knock-down LCs.

We next sought to uncover transcription factor (TF) based regulons orchestrating transcriptional programming of immunoregulation in LCs. Single cell regulatory network inference analysis (SCENIC) in steady state and migrated LCs from the same skin donor identified 16 regulons in the steady state and 26 in migrated LCs (36) (z-score enrichment >0.4, **Figure 4A**, **Supplementary Figures 4A, B**). In agreement with the observed induction of immunocompetence, regulons identified in migrated LCs were reported in immune cell activation states (*JUND, KLF6, STAT1, RELB, IRF4*, **Figure 4A**, **Supplementary Figure 4A**). Similar regulons were identified across multiple donors (*IRF4, KLF6. JUND, RELB*, **Supplementary Figures 4C, D**) To delineate candidate TFs that program immunoregulation in migrated LCs, 5 transcription factors with the highest changes in their gene expression levels were selected for partial information decomposition analysis in context (PIDC) (40), (**Figure 4B**). PIDC is designed and benchmarked for GRN inference from single cell RNA-seq data, and is an extended formalism using multivariate information measures for gene triplets in the context of every cell in the dataset. However, since the information in a GRN flows from TFs to target genes, we restricted the directionality of the edges within the inferred network, including only interaction edges consistent with the information flow (TF -> target gene, directional PIDC). The resulting network comprised 70 edges with weight higher than 1, and when hierarchically organized, predicted distinct regulatory modules for genes in Tol1 programme (**Figure 4C**, **Supplementary Table 5**). Interestingly, directional PIDC network analysis indicated combinatorial regulation of the majority of genes, with a single transcription factor implicated only for 3 targets. Notably, 7 target genes including the transcription factor KLF6 were predicted to be regulated by *IRF4* (**Figure 4C**, edge Importance >1). Given the functions of IRF4 in promoting the tolerogenic programming of murine dendritic cells (26) and our successful CRISPR-Cas9 editing of the IRF4 locus in human LCs and their scRNA-seq profiling (8) we confirmed, that expression of 3 out of the 7 predicted genes was compromised in IRF4 knock-down LCs (**Figure 4D**). PIDC analysis of migrated LC from additional donors inferred *IRF4* regulation of these 3 target genes, as well as *IDO1* (Edge importance values *IDO1*: 1.30, *LGALS1*: 1.09, *IL4I1* (0.89) and *LAMTOR1* (0.55) **Supplementary Table 6**). Thus, LC migration from the epidermis results in a switching of their transcriptional state resulting in the enhanced expression of an immunoregulatory module that is partly dependent on IRF4 and underpins priming of Treg responses.

## Discussion

While LC-mediated immunoregulation appears critical for cutaneous and systemic immune homeostasis, analyses of the genomic programs and molecular pathways that enable human LCs to promote immunoregulatory responses has been hampered by the lack of suitable experimental systems and limitations of available technologies. Here, we analyzed primary human LCs both in steady-state and upon migration from the epidermis for their ability to prime Treg responses. Importantly, these functional analyses were coupled with single-cell transcriptional profiling using Drop-seq (29). Multivariate information measures were then used to predict a transcriptional network underpinning LC immunoregulatory function. This approach revealed that migration out of the epidermis strongly induced LC maturation and immunocompetence. Additionally, transcriptomes of steady-state LCs diverged into two clusters, with a gradation of immunocompetence. Importantly, the transcriptional states of *in vitro* migratory LCs matched recently published transcriptomes of steady-state LCs (11, 38), including expression of *EPCAM*, a classical *in situ* LC marker, and indicated that both S1 and S2 clusters represented activated cells, based on the expression of *PIM3*, *MMP9*, *LMNA* and *STK17B*.

Classically, the ability of APCs to suppress immune responses thereby inducing tolerance has been associated with their immature state characterized by lower expression of the antigen processing and presentation machinery and co-stimulatory molecules (19, 20). Indeed, earlier studies have shown that DCs in an immature state, expressing low levels of antigen presenting and co-stimulatory molecules, can drive tolerogenic responses by inducing anergy of antigen-specific T cells and expanding Tregs (18, 16). In contrast, here we demonstrate that such immature LCs inefficiently induce Tregs, while a specific subset of immunocompetent LCs expressing CD86 are able to do so more effectively. Supporting the linkage between immunocompetence and ability to prime immunoregulatory responses, LC migration out of the epidermis resulted in a further increase in their ability to induce Tregs when compared with the immunocompetent (S2) steady-state LCs. This observation is corroborated by recent findings, suggesting overlap between migratory and immunoregulatory DC transcriptional programmes, that function during homeostasis to keep in check responses to self-antigens in peripheral tissues (41). The observation that maturation is coupled with ability to induce tolerogenic T cells strongly converges with our earlier studies using a model system of murine bone-marrow derived DCs to analyse the transcriptional and epigenomic control of tolerogenic responses (26). It was demonstrated in that system that tolerogenic functions are up-regulated during DC maturation, *via* the transcription factor IRF4, which functions in part to dampen inflammatory cytokine signalling. In a striking parallel, we have recently shown that IRF4 is up-regulated upon migration of human LCs and it functions to repress oxidative stress and inflammatory cytokine signalling gene modules (8). Thus, in totality these findings strongly suggest that in both murine and human APCs, the transcription factor IRF4, which is upregulated during migration, functions as a regulatory determinant to couple efficient antigen presentation with immunoregulatory programming of T cell responses.

LC-specific immunoregulatory programme comprised 30 genes, including those encoding classic markers of immunotolerance (such as *IDO1*, *IDO2*, *CD274*) and genes encoding LC maturation (CD80, CD83, CD86). This programme was shared with other skin-resident DC populations, such as CD14/CD141- expressing cells and skin-resident MoDC populations [**Supplementary Figures 2H–K** (5, 38)], highlighting its generality, and the importance of functional conservation of transcriptional programmes between LCs and other skin DC populations (5, 8). Interestingly, the programme was expressed strongest in LCs, indicating their immunoregulatory competence. The increased expression of T cell co-stimulatory genes, such as CD86 in the steady-state (immunocompetent) and migrated LCs, are suggestive of their importance in Treg induction. Indeed, CD86 activity has been implicated in DC-mediated tolerance induction through interaction with CTLA4 on T cells (42, 43). However, since LC ability to induce immunoregulatory responses is substantially enhanced upon their migration out of the epidermis, it is likely to be governed by additional factors beyond the capacity to process and present antigens and interact more effectively with T cells *via* co-stimulatory molecules. Consistent with this possibility, we observed inducible expression of the immunoregulatory gene module, including *IDO1*, *LAMTOR1, IL4L1* and *LGALS1* across LC populations. While specific genes were present in select LCs in the steady state, both investigated by us and reported by others (38) (**Supplementary Figures 2I–K**), their expression greatly increased on migration. *IDO1* is a classical immunoregulatory mediator, which catabolizes tryptophan leading to skewing of T cell differentiation towards Tregs (44). Galectin-1 encoded by the *LGALS1* gene has been shown to promote the generation of tolerogenic DCs and to enable Tr1 type Tregs to suppress Th1- and Th17-mediated inflammation (45, 45). Thus, Galectin-1 secreted by LCs could function in an autocrine as well as paracrine manner to promote Treg responses. The enzymes *IL4I1*, a mediator of H_2_O_2_ production and *HMOX1*, which degrades Heme, have been shown to be expressed by DC and are implicated in the suppression of effector T cell activation and the induction of Tregs (46–49). Additionally, *LAMTOR1* is implicated in macrophage polarization towards an immunoregulatory M2 phenotype (50).

Interestingly, PIDC analyses suggests a combinatorial set of TFs that may orchestrate the immunoregulatory transcriptional programme in LCs. This set includes IRF4 (8, 26), KLF6 (51), RELB (52, 53) and ELK1 (54) that have been previously implicated in regulation of immune functions in multiple contexts. In contrast, HMGN3 binds to nucleosomes and regulates chromatin organization (55). Thus, its predicted function in LCs is intriguing, and warrants further investigation.

Importantly, analyses of CRISPR-Cas9 mediated knock-down of IRF4 in LCs confirmed the dependence of several immunoregulation-related genes, including *IL4L1* and *LGALS1* and *LAMTOR1* on IRF4. Interestingly, IRF4 did not seem to directly regulate expression of IDO1. However, a recent study demonstrated that IRF4 can form a multipartite transcriptional complex with AHR, a well-established inducer of IDO1 (56, 57), that binds to promoter elements of immunoregulation associated genes (58). High levels of IRF4 expression could thus potentially promote more efficient AHR action, and an increase in IDO1 expression. Induction of *AHR, IRF4* and *IDO1* axis upon migration provides a mechanism for inducible IDO1 expression upon activation and suggests the existence of a positive feedback loop for promoting immunoregulatory responses. This has been previously observed in other DCs, where kynurenine metabolites, produced during IDO-mediated catabolism of tryptophan, feedback to AHRs to sustain IDO expression (59, 56)

The ability of mature LCs to induce immunoregulatory T cell responses appears to be seemingly be at odds with their superiority to induce efficient cytotoxic CD8 T cell responses, through antigen cross-presentation and IL15 release, reported by us and others (6–8, 25), as well as with their ability to prime Th2 and Th17 helper T cells (6, 60). When considering regulation of T cell responses by dendritic cells, two mutually non-exclusive models have been proposed: one model postulates specialized DC subsets that are dedicated for the induction of specific T cell responses e.g. induction of CD8T cells by IRF8+ DCs vs enhanced priming of CD4 T cell responses by IRF4+ DCs (26). In a contrasting model, particular DCs can have sufficient flexibility to induce divergent T cell responses that are dictated by the inflammatory context and cytokine milieu (61–63). Research by us and others indicates that LC indeed display considerable degree of plasticity, likely due to their unique functional roles in the epidermis and transcriptional programming that in the steady state is dependent on IRF4 but not IRF8 expression (8, 28). Importantly, the differential outcome of LC function can be induced by signals emanating from the microenvironment. By analyzing molecular regulatory circuits across LCs and other DC types we proposed that immunoregulatory and immunogenic responses of LCs are directed by signaling from the epidermis and involve counter-acting gene circuits that are coupled to a core maturation gene module regulated by NFkB, and two members of IRF family: IRF4 and IRF1 (28). Our model proposes, that while epidermal signaling in the steady-state promotes LC immunoregulatory function, the disruption of cell-cell contacts coupled with inflammatory signaling induces LC immunogenic programming, *via* IRF1 and IRF1-dependent transcriptional programmes (27, 64). During inflammation, with exposure to proinflammatory cytokines, such as TNF, LCs ability to stimulate CD8 T cells increases significantly. LC-derived IL15 has been shown to specifically promote activation and expansion of CD8 T cells (65–67). Given these findings it is highly plausible that LCs promote both regulatory and cytotoxic T cell responses, with the timing and balancing of these counteracting functions contributing to the potency of immunogenic responses and their eventual resolution in the epidermis.

It is important to highlight, that our experiments were conducted *ex vivo*, using LCs isolated from the skin microenvironment. While this system allows investigations of human LCs, it presents its own limitations. As the *ex vivo* manipulation of LCs (digestion, *in vitro* culture, etc.) might have led to changes not reflective of what happens *in vivo*, further research in an appropriate *in vivo* model is required to confirm the identified immunoregulatory transcriptional networks in LCs in the steady-state. Our analysis of LC functional ability to regulate T cell activation *via* generation of Tregs was limited to migrated LCs, as the strongest expressors of immunoregulatory programme. These LCs partially inhibited CD4 T cell proliferation, suggesting the need for more complex signalling inputs, or the limitations of our experimental conditions i.e., the mixture of cells used and/or short assay time.

Our analyses document that efficient priming of immunoregulatory responses by LCs critically requires upregulation of a migration-coupled maturation program superimposed with a immunoregulation-inducing gene module. While the induction of this immunoregulatory programme in LCs is complex, IRF4 is likely to act as a pivotal switch regulating LC immune function and orchestrating complementary modules in LC transcriptional programming. The enhancement of LC immunoregulatory abilities on maturation could be explored therapeutically to reinstate tolerance in the skin during inflammatory conditions.

## Data availability statement

The RNA-sequencing data are deposited at GEO under accession number GSE142298, https://www.ncbi.nlm.nih.gov/geo/query/acc.cgi?acc=GSE142298.

## Ethics statement

The studies involving human participants were reviewed and approved by South East Coast - Brighton & Sussex Research Ethics Committee in adherence to Helsinki Guidelines [ethical approvals: REC approval: 16/LO/0999). The ethics committee waived the requirement of written informed consent for participation.

## Author contributions

MP, SS, JD, HS: intellectually conceived and wrote the manuscript, planned the experiments and analysed the results. SS, JD, KC, GD, AV: run functional experiments, flow cytometry, single-cell sequencing. AV, JD, PSK, JW: developed and optimized scRNA-sequencing. JD, BMA, AV, PSK, MP: analysis and meta-analysis of scRNA-seq data. JD, GD, BMA, MP reconstruction and modelling of gene regulatory networks. MP, MA-J, HS: discussions, data analysis, reviewing of the manuscript. All authors contributed to the article and approved the submitted version.

## Funding

The study was funded by a Sir Hendy Dale Fellowship from Wellcome Trust, 109377/Z/15/Z. Development of single cell Drop-Seq technology was funded by MRC grant MC_PC_15078. This project in the Pitt Center for Systems Immunology was supported by UPMC-ITTC funds.

## Acknowledgments

We are grateful to the subjects who participated in this study. We would like to thank Prof Peter Friedmann for in-depth review of the manuscript. We acknowledge the use of the IRIDIS High Performance Computing Facility and Flow Cytometry Core Facilities, together with support services at the University of Southampton.

## Conflict of interest

The authors declare that the research was conducted in the absence of any commercial or financial relationships that could be construed as a potential conflict of interest.

## Publisher’s note

All claims expressed in this article are solely those of the authors and do not necessarily represent those of their affiliated organizations, or those of the publisher, the editors and the reviewers. Any product that may be evaluated in this article, or claim that may be made by its manufacturer, is not guaranteed or endorsed by the publisher.
