# Supplementary material for: Transcriptional programming of immunoregulatory responses in human Langerhans cells
Source: Front Immunol. 2022 Sep 20;13:892254. doi: 10.3389/fimmu.2022.892254 (PMC9530347; doi:10.3389/fimmu.2022.892254)
Supplement: Supplementary file 1 [file DataSheet_1.pdf]

Supplementary Materials for

# Transcriptional programming of immunoregulatory responses in human Langerhans cells

James Davies†, Sofia Sirvent†, Andres F. Vallejo, Kalum Clayton, Gemma Porter, Patrick Stumpf, Jonathan West, Michael Ardern-Jones, Ben MacArthur, Harinder Singh* and Marta E Polak*

*Corresponding author. Email: m.e.polak@soton.ac.uk
harinder@pitt.edu

**This PDF file includes:**

Supplementary Figures S1 to S4
Captions for Supplementary Tables S1 to S6

**Supplementary Figure S1.**

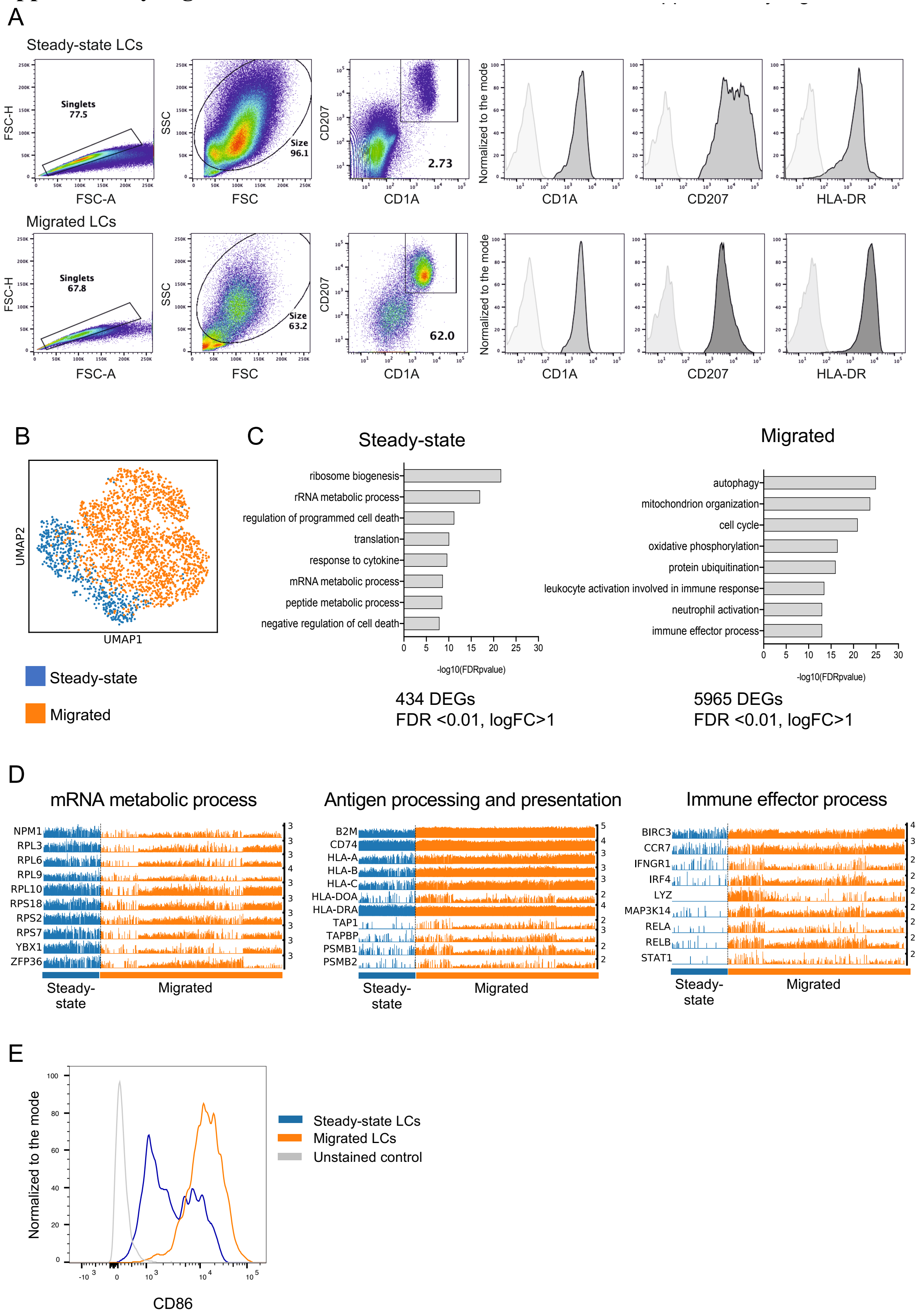

**Supplementary Figure S1.**

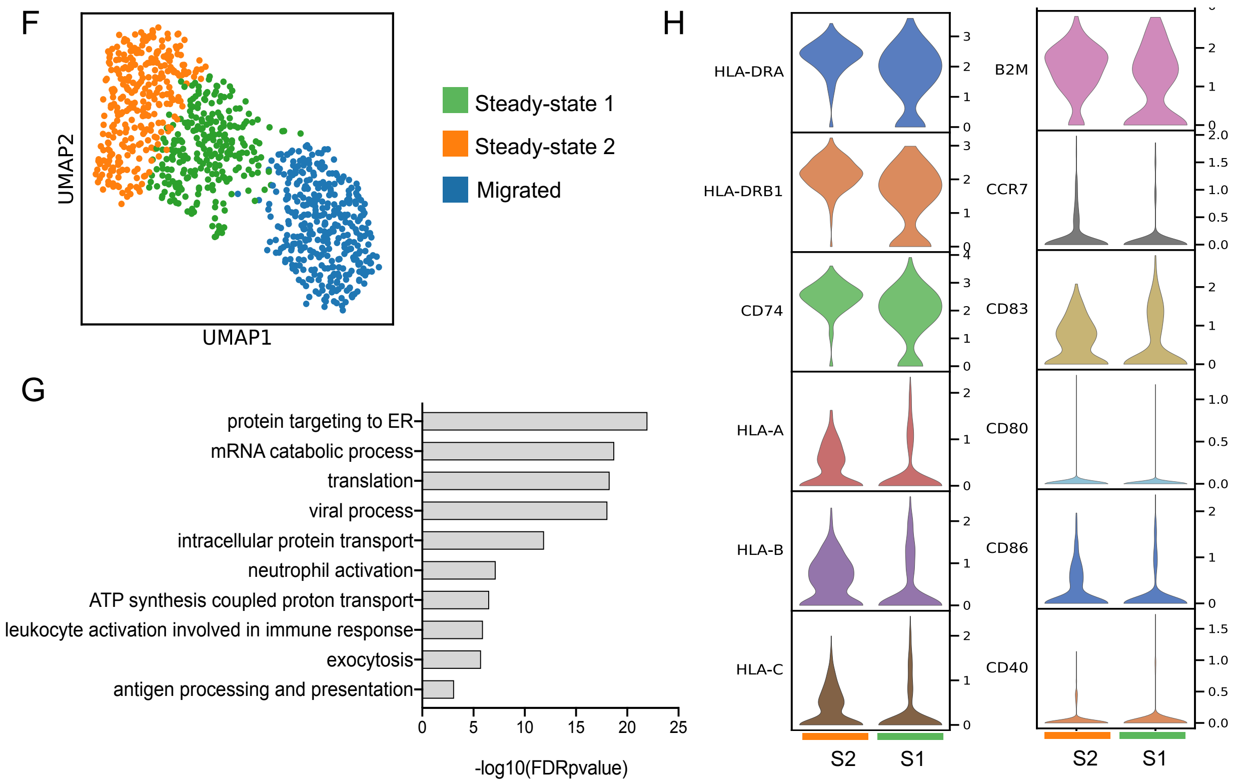

**Supplementary Figure S1.**

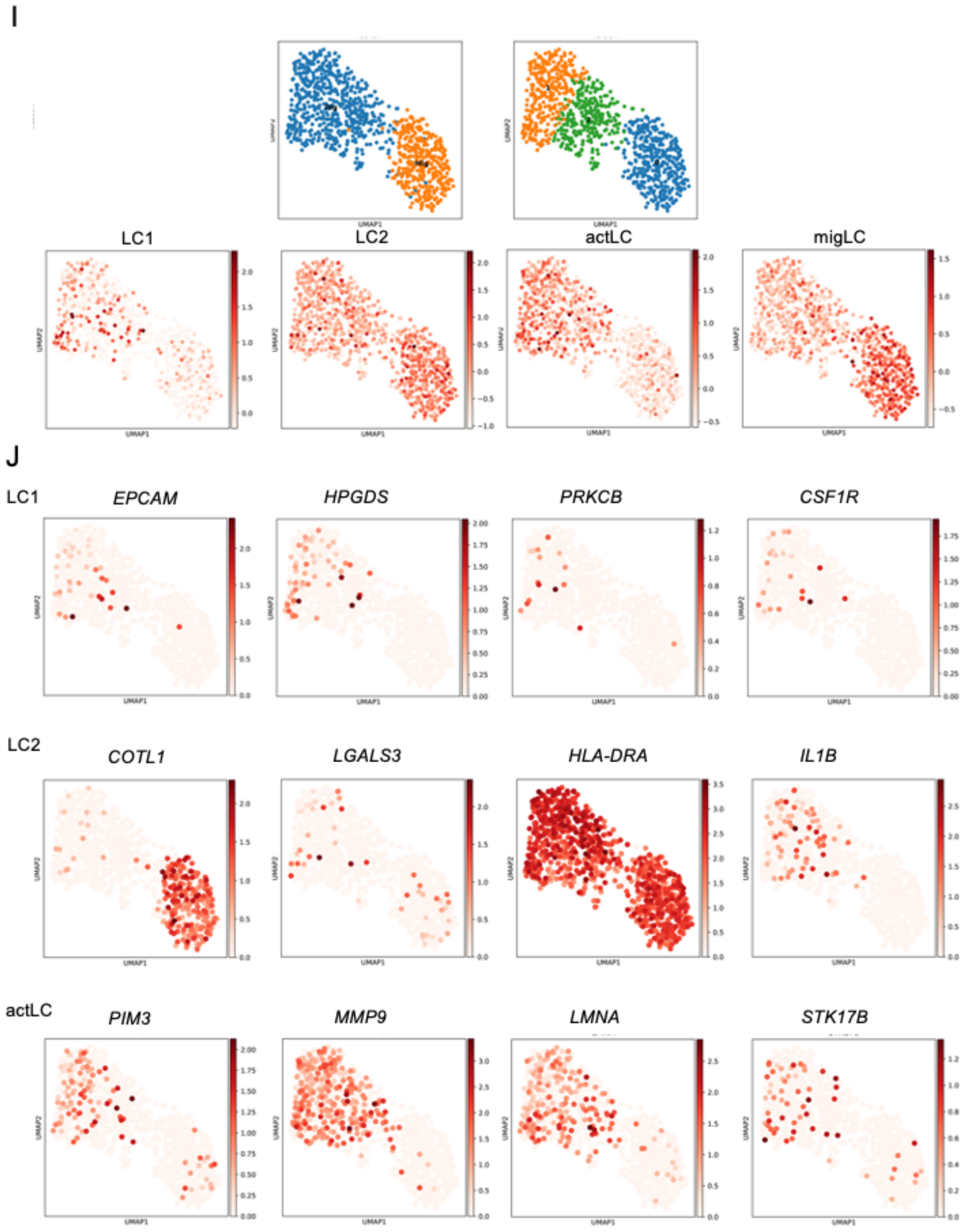

**Supplementary Figure S1.**
**LC migration from the epidermis induces immunocompetence associated transcriptional modules.**

**A.** Gating strategy for isolation of steady state and migrated Langerhans cells from human skin. **B.** UMAP dimensionality reduction analysis of Scran normalised single cell data from steady-state and migrated LCs from Figure 1 (donor D1), along with 3 additional previously processed migrated LC datasets (donors D2-D4). **C.** Gene ontology analysis (Toppgene) results for the combined DEG analysis of steady-state (434 upregulated DEGs) and migrated LC (5965 upregulated DEGs) (-log10 FDR corrected p-values) as displayed in Figure S1A. **D.** Trackplots displaying genes included in ontologies upregulated in steady-state (mRNA metabolic process) and migrated LC (antigen processing and presentation, immune effector process). **E.** Expression levels of LC activation marker, CD86, in steady state (blue) and migrated (orange) LCs as measured by flow cytometry. Grey histogram depicts negative staining control. **F.** UMAP marker plot as in Figure 1A, with leiden clustering (r=0.5) displaying subpopulations S1 and S2 amongst the steady-state LC population. **G.** Gene ontology analysis (Toppgene) results for the 372 upregulated DEGs in S2 steady-state LCs (-log10 FDR corrected p-values). **H.** Violin plots displaying the expression of MHC I, MHC II and co-expression molecules amongst S1 and S2 steady-state LCs. **I.** Marker plots displaying the average expression (z-scores) of LC1, LC2, actLC and migLC signature genes from Liu et al. **J.** Marker plots displaying the individual gene expression values of genes within LC1, LC2, actLC and migLC signatures from Liu et al.

**Supplementary Figure S2.**

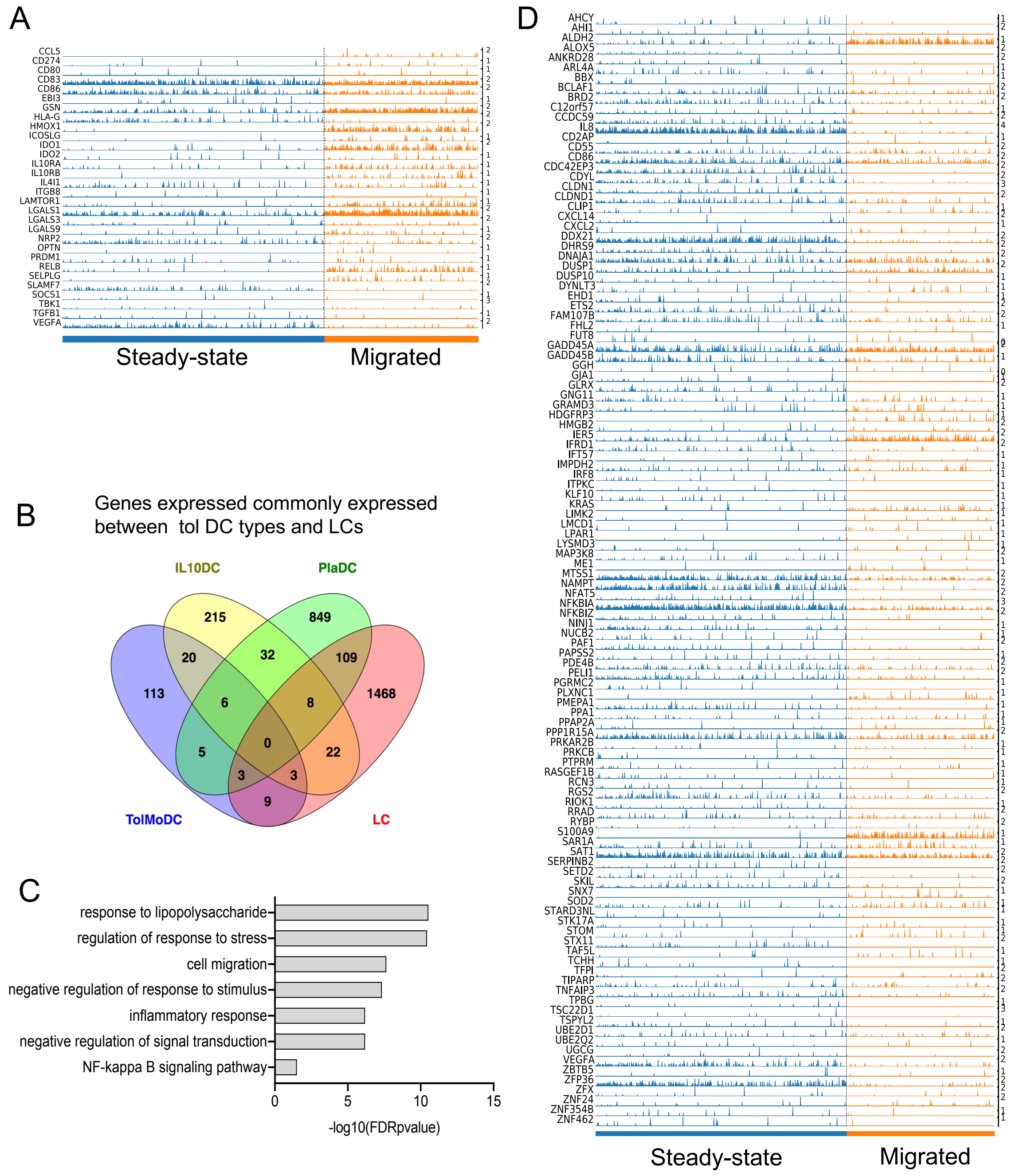

**Supplementary Figure S2.**

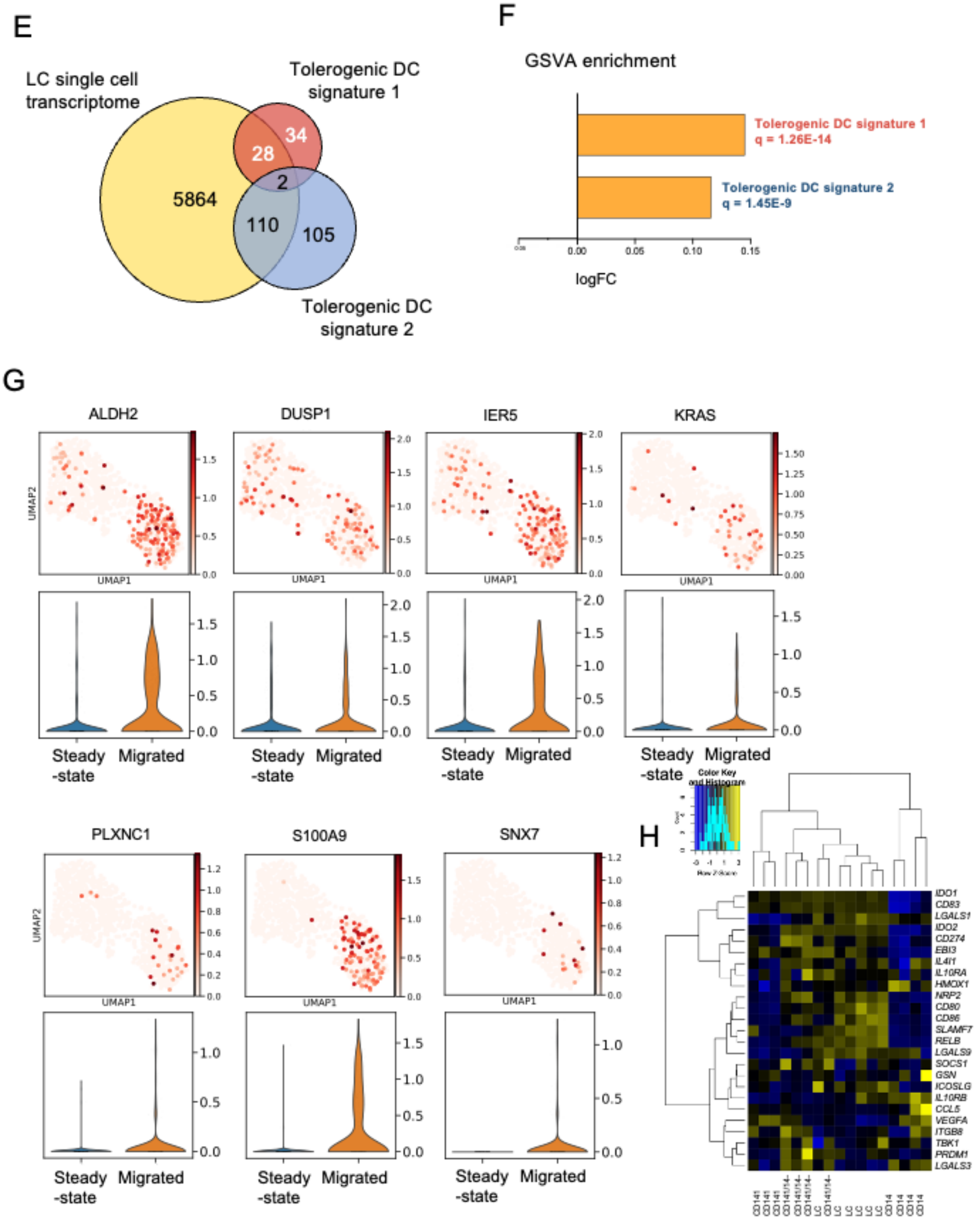

**Supplementary Figure S2.**

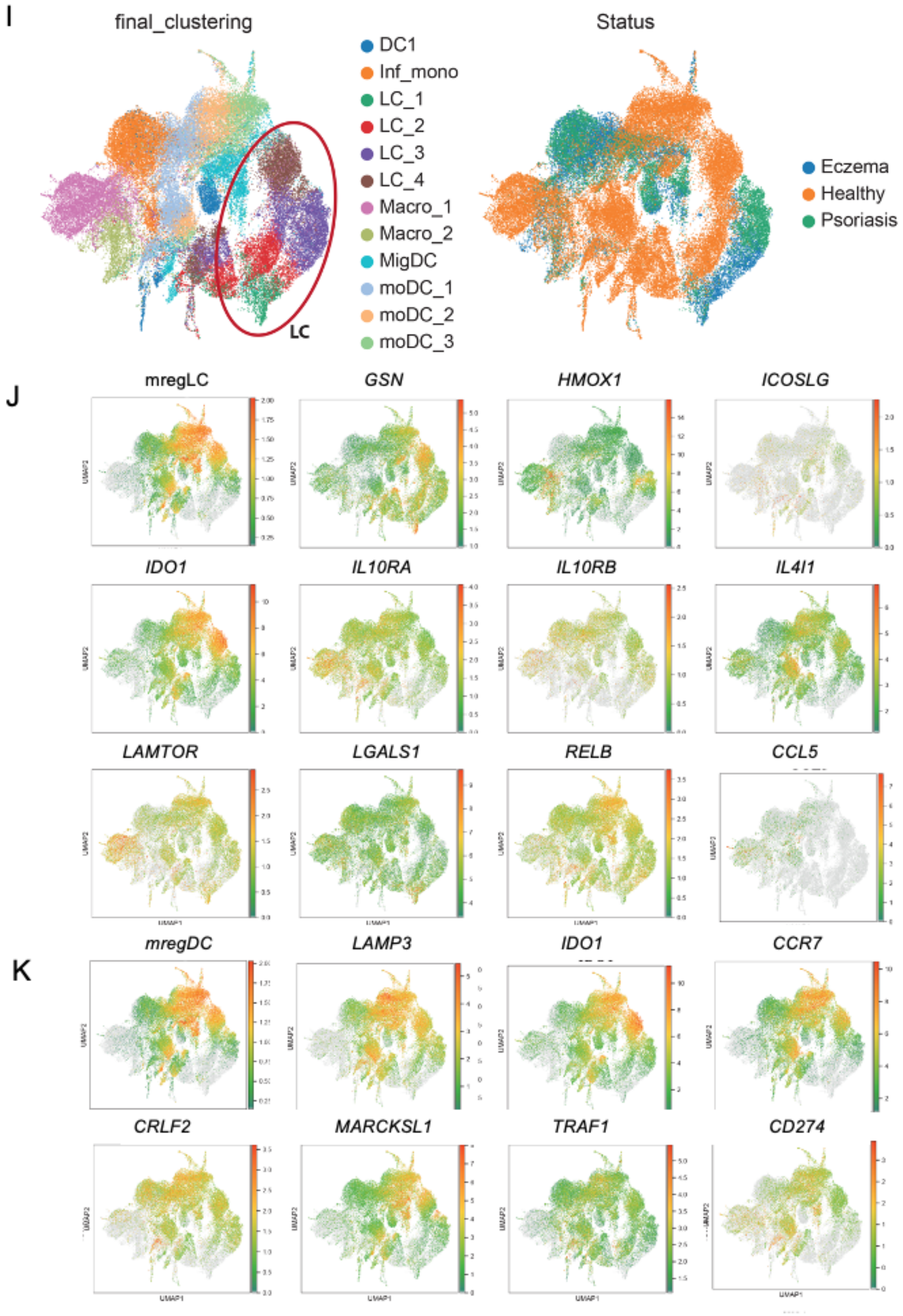

**Supplementary Figure S2.**

**Migration of LCs from the epidermis enhances their immunoregulatory transcriptional programming.**

**A.** Trackplot of all 30 genes from the tol 1 signature which are within the whole LC single cell transcriptomic dataset. **B.** Venn Diagram displaying the overlap in upregulated DEGs (Limma, FDR corrected p-value<0.05, logFC>1), identified during comparison between steady-state LC-MoDC (1622 genes, GSE23618), PlaDC-MoDC (1012 genes, GSE52850), TolMoDC-MoDC (160 genes, GSE52894) and IL10MoDC-MoDC (306 genes, GSE117946) in each respective dataset. Tolerogenic gene signature 2 (tol 2) was compiled from genes co-upregulated in two or more of the tolerogenic DC conditions. **C.** Gene ontology analysis (Toppgene) for the tolerogenic gene signature 2 (217 genes) which were co-upregulated in two or more of the tolerogenic DC conditions from Figure S2C (-log10adj.p-values). **D.** Trackplot of all 112 genes from the tol 2 signature which are within the whole LC single cell transcriptomic dataset. **E**. Venn diagram displaying the number of genes from tolerogenic gene signature 1 (tol 1, total=64) and tolerogenic gene signature 2 (tol 2, total=217) within the whole LC single cell dataset. **F**. Gene Set Variation Analysis (GSVA) displaying enrichment of tol 1 and tol 2 in the LC populations. FDR corrected p-values and logFC are displayed. **G.** Violin plots and UMAP marker plots displaying the expression of genes within tol 2 amongst steady-state and migrated LCs (FDR corrected p-values <0.01, logFC>1). **H.** Heatmap plot displaying the normalised expression of 25/30 Tolerogenic DC signature 1 genes expressed in LC single cell RNA-seq data, detected in microarray data (GSE66355) of migratory LC and DDC subpopulations (CD14+, CD141+ and CD14-CD141). **I – K.** Re-analysis of antigen presenting cells subsetted from publicly available data from Human Skin Atlas (HSA) **I.** Unbiased clustering of antigen presenting cells in UMAP space (Leiden r=0.5), left: colors denote antigen presenting cells as defined in HSA, right: colors denote skin disease status, eczema (blue), psoriasis (green), healthy(orange). **J** Expression of LC genes in tol1 signature across skin antigen presenting cell populations. First UMAP represents Z-scores for Tol1 signature, following UMAPs depict individual gene expression values, normalized gene expression shown. **K** Expression of mregDC genes across skin antigen presenting cell populations. First UMAP represents Z-scores for mregDC signature, following UMAPs depict individual gene expression values, normalized gene expression shown.

**Supplementary Figure S3.**

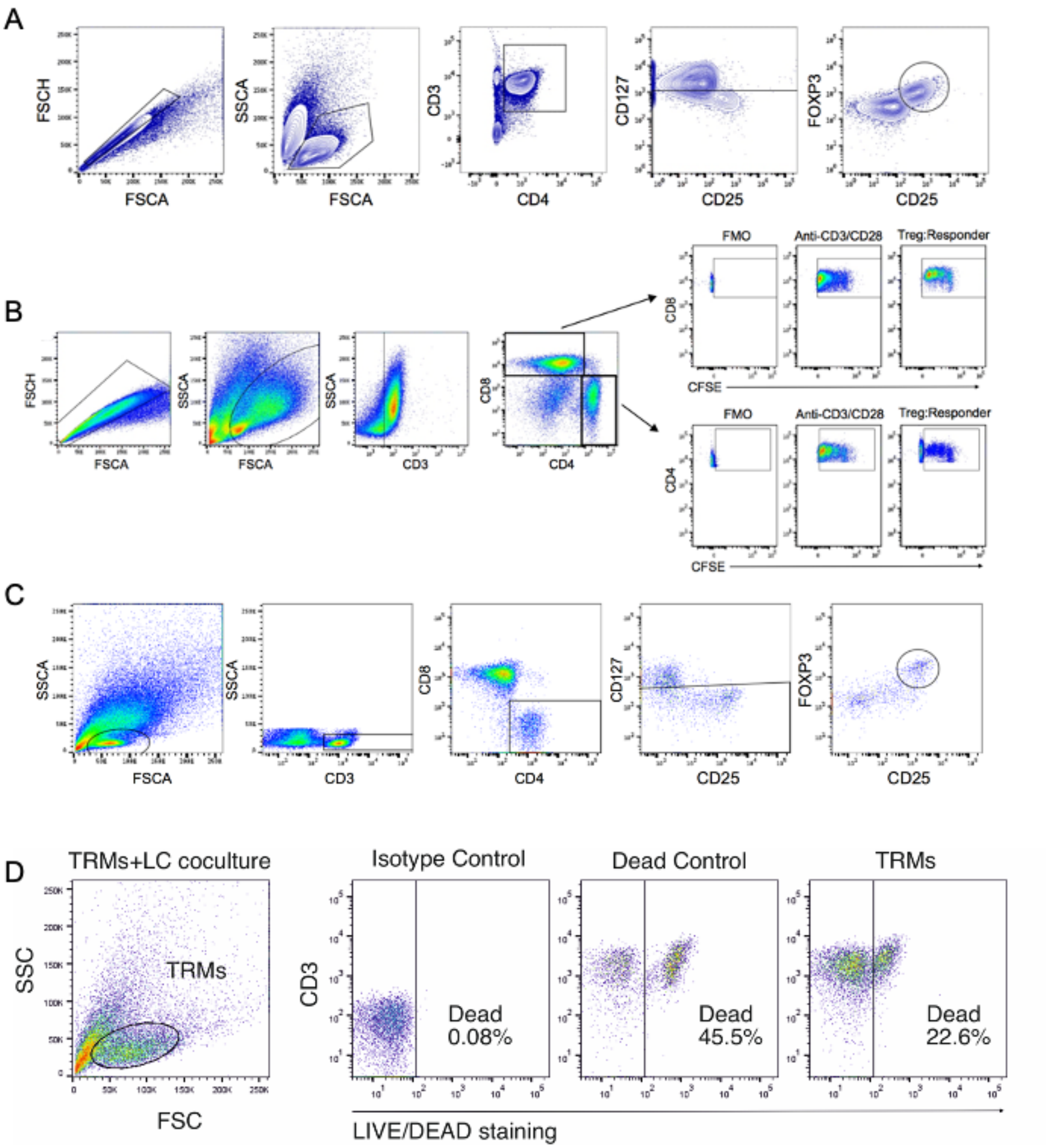

**Supplementary Figure S3.**

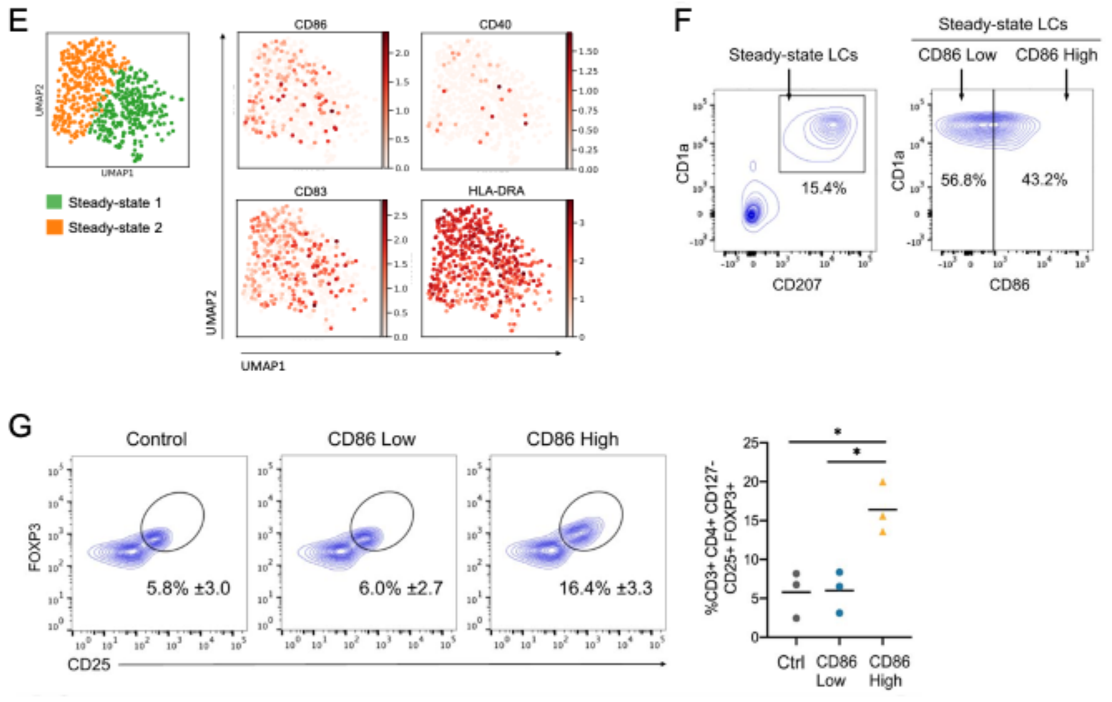


**Supplementary Figure S3.**

**Migrated LCs more efficiently prime functional Treg responses**

**A.** Gating strategy for investigating the quantity of CD3+CD4+CD127-CD25+FOXP3+ Tregs after co-culture of CD4 naïve T cells with LC for 5-days. **B.** Gating strategy for investigating CFSE labelled PBMC proliferation, selecting for CD4+ and CD8+ T cell populations. CFSE measurement gating was applied to responder cell population only, excluding unlabelled CFSE negative Tregs. **C.** Gating strategy for investigating the quantity of Tregs induced after co-culture of autologous TRMs with migrated LC for 5-days. **D.** Gating strategy for live TRM. **E.** UMAP projection displaying S1 and S2 LC with the expression of CD86, CD40, CD83 and HLA-DRA shown. **E.** Flow cytometry assessment of steady-state LCs identified as CD207/CD1a high cells. LC populations were separated into CD86Low and CD86High by FACS. Representative example from n=3 independent LC donors. **G**. Flow cytometry assessment of CD4+ naive T cells after 5-day co-culture with either CD86Low or CD86High steady-state LC. 5-day cultures of CD4+ naïve T cells alone were used as control. Tregs were identified as CD3+CD4+CD127-CD25+FOXP3+ cells. n=3 independent LC donor paired experiments. *$p<0.05$.

**Supplementary Figure S4.**

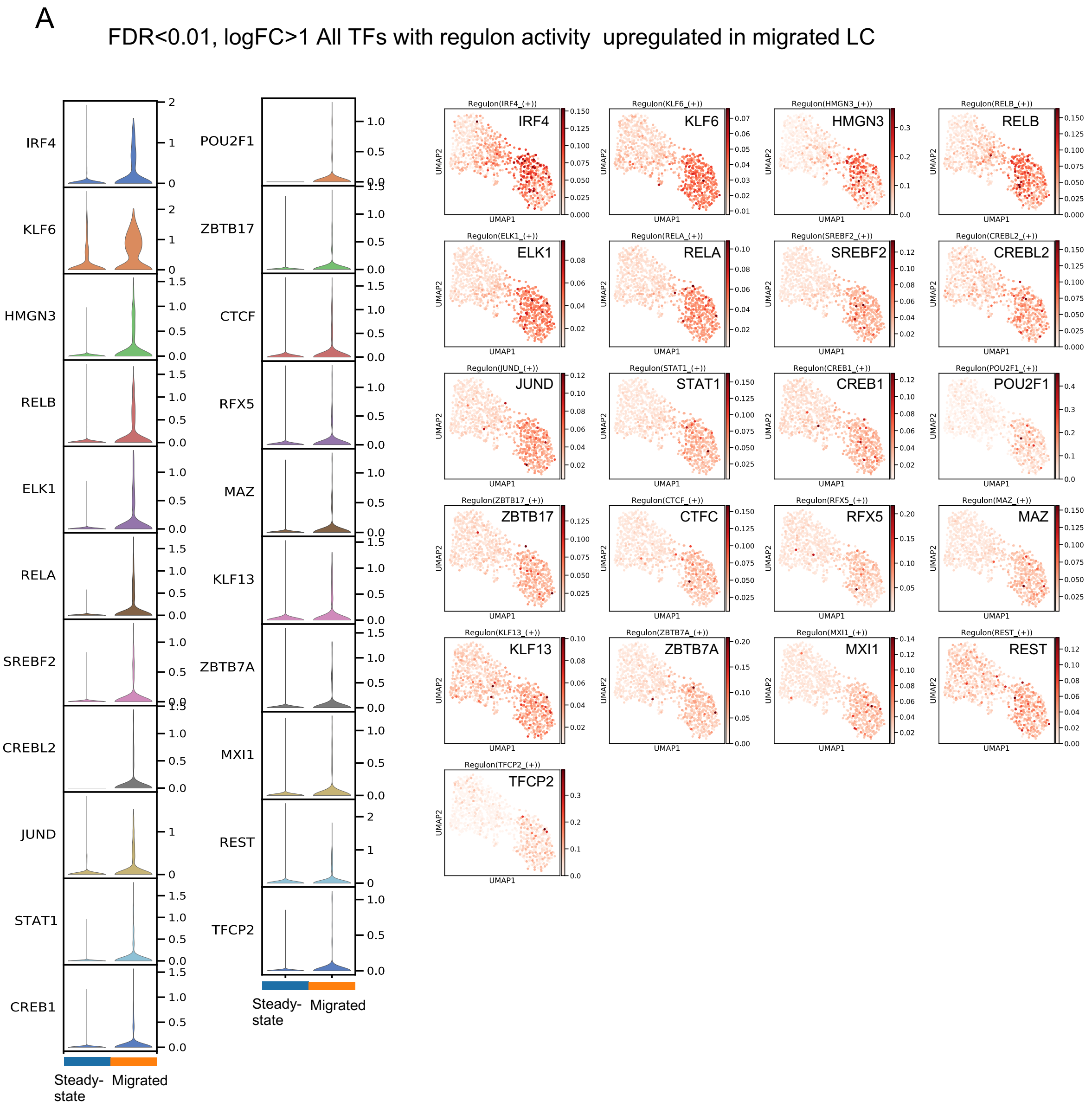

B

FDR<0.01, logFC>1 All TFs with regulon activity upregulated in Steady-state LC

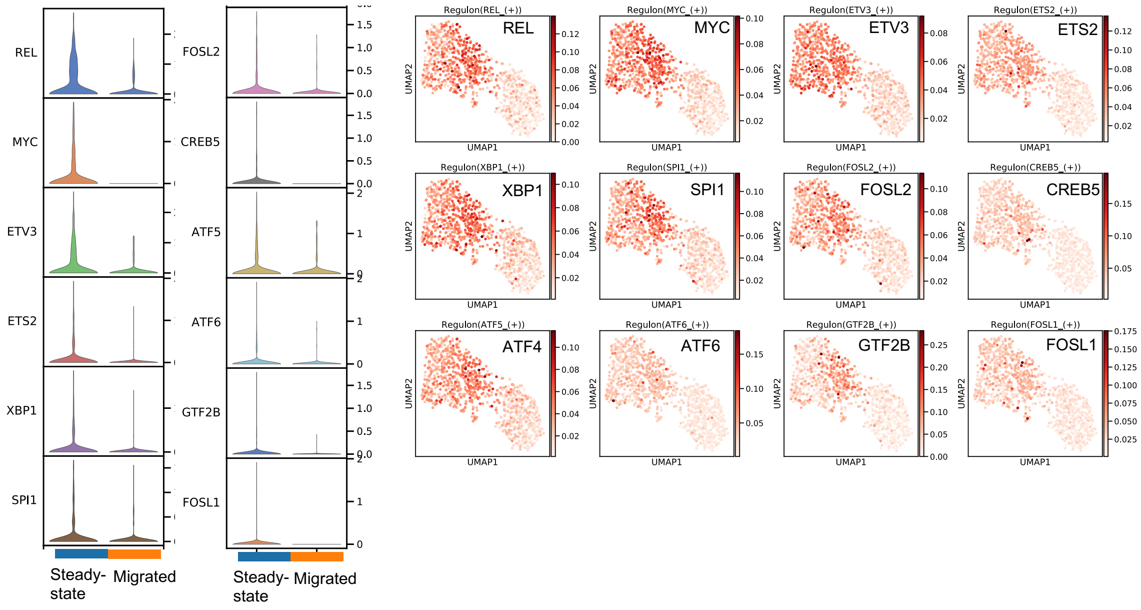


C

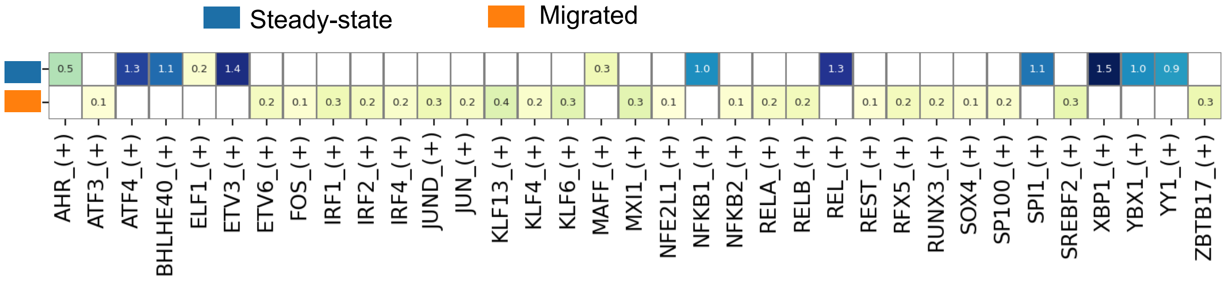


D

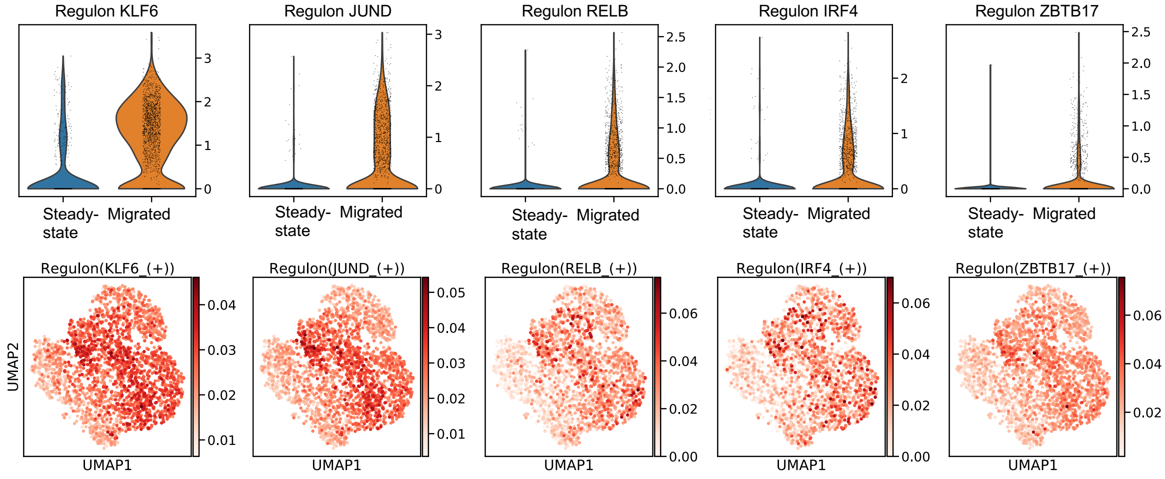

**Supplementary Figure S4.**

**Transcriptional network underlying LC immunoregulatory programming**

**A.** Violin plots displaying the transcriptomic expression of migrated LC upregulated TFs (FDR corrected p-values<0.01, logFC>1) identified to be enriched in migrated LCs from SCENIC analysis (z-score>0.4). UMAP marker plots showing TF regulon enrichment Z-scores in each cell, across the two LC populations are displayed alongside. **B**. Violin plots displaying the transcriptomic expression of steady-state LC upregulated TFs (FDR corrected p-values<0.01, logFC>1) identified to be enriched in steady-state LCs from SCENIC analysis (z-score>0.4). UMAP marker plots showing TF regulon enrichment Z-scores in each cell, across the steady-state and migrated LC populations are displayed alongside. **C.** SCENIC regulatory network and inference clustering analysis from combined steady-state and expanded migrated LC dataset analysis (Datasets D1-D4 as in Figure S1A) revealed TF regulons which were enriched in steady-state and migrated LCs. Z-score heatmap of enriched regulons are displayed (z-score>0.1). **D.** Violin plots displaying the transcriptomic expression of TFs identified to be enriched in migrated LCs from SCENIC analysis of the combined LC dataset (Figure S4C). UMAP marker plots showing TF regulon enrichment Z-scores in each cell, across the LC populations are displayed.

## Supplementary Tables – Captions

**Supplementary Table S1.**

**Differentially expressed genes: Steady-state vs migrated LCs**

DEGs comparing steady-state and migrated LCs (within D1 only and steady-state vs migrated LC in D1-D4) using T-test within Scanpy (FDR corrected p-value<0.01, logFC>1). Biological pathways and ontologies associated with DEGs were identified in Toppgene (FDR corrected p-value<0.05).

**Supplementary Table S2.**

**Differentially expressed genes: Steady-state 1 vs 2**

DEGs comparing steady-state 1 and 2 using T-test within scanpy (FDR corrected p-value<0.01, logFC>1). Biological pathways and ontologies associated with DEGs were identified in Toppgene (FDR corrected p-value<0.05).

Type or paste caption here.

**Supplementary Table S3.**

**Tolerogenic DC gene signature 1**

Literature reviews and experimental papers referencing genes associated with DC and macrophage tolerogenic function were summarised into a 64 gene signature.

**Supplementary Table S4**

**Tolerogenic DC gene signature 2**.

Signature was compiled of DEGs (Limma, FDR corrected p-value<0.05, logFC>1), identified during comparison between steady-state LC-MoDC (1622 steady-state LC upregulated genes, GSE23618), PlaDC-MoDC (1012 PlaDC upregulated genes, GSE52850), TolMoDC-MoDC (160 TolMoDC upregulated genes, GSE52894) and IL10MoDC-MoDC (306 IL10MoDC upregulated genes, GSE117946) in each respective dataset, that were co-upregulated in two or more of the tolerogenic DC conditions.

**Supplementary Table S5**

**PIDC network**

List of edges for directional PIDC, donor 1. SCRAN-normalised data for migrated LCs including genes from tol1 signatures and selected transcription factors was used for network inference using PDIC algorithm

**Supplementary Table S6**

**PIDC network 2**

List of edges for directional PIDC, donors 2-4, SCRAN-normalised data for migrated LCs including genes from tol1 signatures and selected transcription factors was used for network inference using PDIC algorithm
